# Survival prediction of hepatocellular carcinoma by measuring the extracellular volume fraction with single-phase contrast-enhanced dual-energy CT imaging

**DOI:** 10.3389/fonc.2023.1199426

**Published:** 2023-07-19

**Authors:** Yan Chen, Kexin Shi, Zhen Li, Huixia Wang, Nana Liu, Pengchao Zhan, Xing Liu, Bo Shang, Ping Hou, Jianbo Gao, Peijie Lyu

**Affiliations:** ^1^ Department of Radiology, The First Affiliated Hospital of Zhengzhou University, Zhengzhou, Henan, China; ^2^ Department of Clinical Medicine, Henan Medical School of Zhengzhou University, Zhengzhou, Henan, China; ^3^ Department of Interventional Radiology, The First Affiliated Hospital of Zhengzhou University, Zhengzhou, Henan, China

**Keywords:** multidetector computed tomography, dual-energy, hepatocellular carcinoma, extracellular volume, prognosis factors

## Abstract

**Purpose:**

This study aimed to investigate the value of quantified extracellular volume fraction (fECV) derived from dual-energy CT (DECT) for predicting the survival outcomes of patients with hepatocellular carcinoma (HCC) after transarterial chemoembolization (TACE).

**Materials and methods:**

A total of 63 patients with HCC who underwent DECT before treatment were retrospectively included. Virtual monochromatic images (VMI) (70 keV) and iodine density images (IDI) during the equilibrium phase (EP) were generated. The tumor VMI-fECV and IDI-fECV were measured and calculated on the whole tumor (Whole) and maximum enhancement of the tumor (Maximum), respectively. Univariate and multivariate Cox models were used to evaluate the effects of clinical and imaging predictors on overall survival (OS) and progression-free survival (PFS).

**Results:**

The correlation between tumor VMI-fECV and IDI-fECV was strong (both *p*< 0.001). The Bland–Altman plot between VMI-fECV and IDI-fECV showed a bias of 5.16% for the Whole and 6.89% for the Maximum modalities, respectively. Increasing tumor VMI-fECV and IDI-fECV were positively related to the effects on OS and PFS (both *p*< 0.05). The tumor IDI-fECV-Maximum was the only congruent independent predictor in patients with HCC after TACE in the multivariate analysis on OS (*p* = 0.000) and PFS (*p* = 0.028). Patients with higher IDI-fECV-Maximum values had better survival rates above the optimal cutoff values, which were 35.42% for OS and 29.37% for PFS.

**Conclusion:**

The quantified fECV determined by the equilibrium-phase contrast-enhanced DECT can potentially predict the survival outcomes of patients with HCC following TACE treatment.

## Introduction

Hepatocellular carcinoma (HCC), which accounts for 75%–85% of primary liver cancer, is the sixth most common cancer and the second most common cause of death in the world (1). Approximately 80% of HCC patients have a history of chronic hepatitis and/or cirrhosis, and 70% of HCC patients at the time of diagnosis have lost the opportunity for curative surgery (2, 3). According to the Barcelona Clinic Liver Cancer (BCLC) system, transarterial chemoembolization (TACE) is the first-line treatment for the intermediate stage of HCC. Some studies also used TACE to treat patients with early and advanced stage HCC (4). TACE can occlude the tumor’s feeding arteries, improve the survival rate of HCC patients with unresectable tumors, and reduce the recurrence of resectable HCC (5, 6). However, a portion of patients with HCC did not respond to the first TACE, and their prognosis was poor. For these patients, a timely conversion to sorafenib or lenvatinib may prevent further liver damage and prolong overall survival (7). Given this, if an accurate effect prediction of the TACE can be made before treatment for these patients, it is possible to refine treatment strategies, identify patients who would benefit most from the TACE, and improve overall treatment outcomes.

In previous studies (8–11), dual-energy CT (DECT) scanning has been proven to be effective and quantitative in evaluating the prognosis of HCC patients after treatment. At the same time, other relevant studies have shown that extracellular volume fraction (fECV) based on the iodine density of DECT scanning can quantify the liver extracellular volume and early identify patients with high risk for developing liver-related adverse events (12–15). However, this application has not yet been investigated for predicting the survival outcomes of HCC patients after TACE.

Given that the fECV derived from DECT can be used to predict the degree of liver cirrhosis quantitatively, we hypothesized that the fECV derived from DECT could be correlated with the survival outcomes of HCC patients. Thus, the purpose of this study was mainly to evaluate the feasibility of fECV quantified by DECT for predicting the survival outcomes of HCC patients after TACE.

## Materials and methods

### Patient characteristics

A waiver of written informed consent was granted by our institutional review board for this retrospective study.

**Figure 1:**
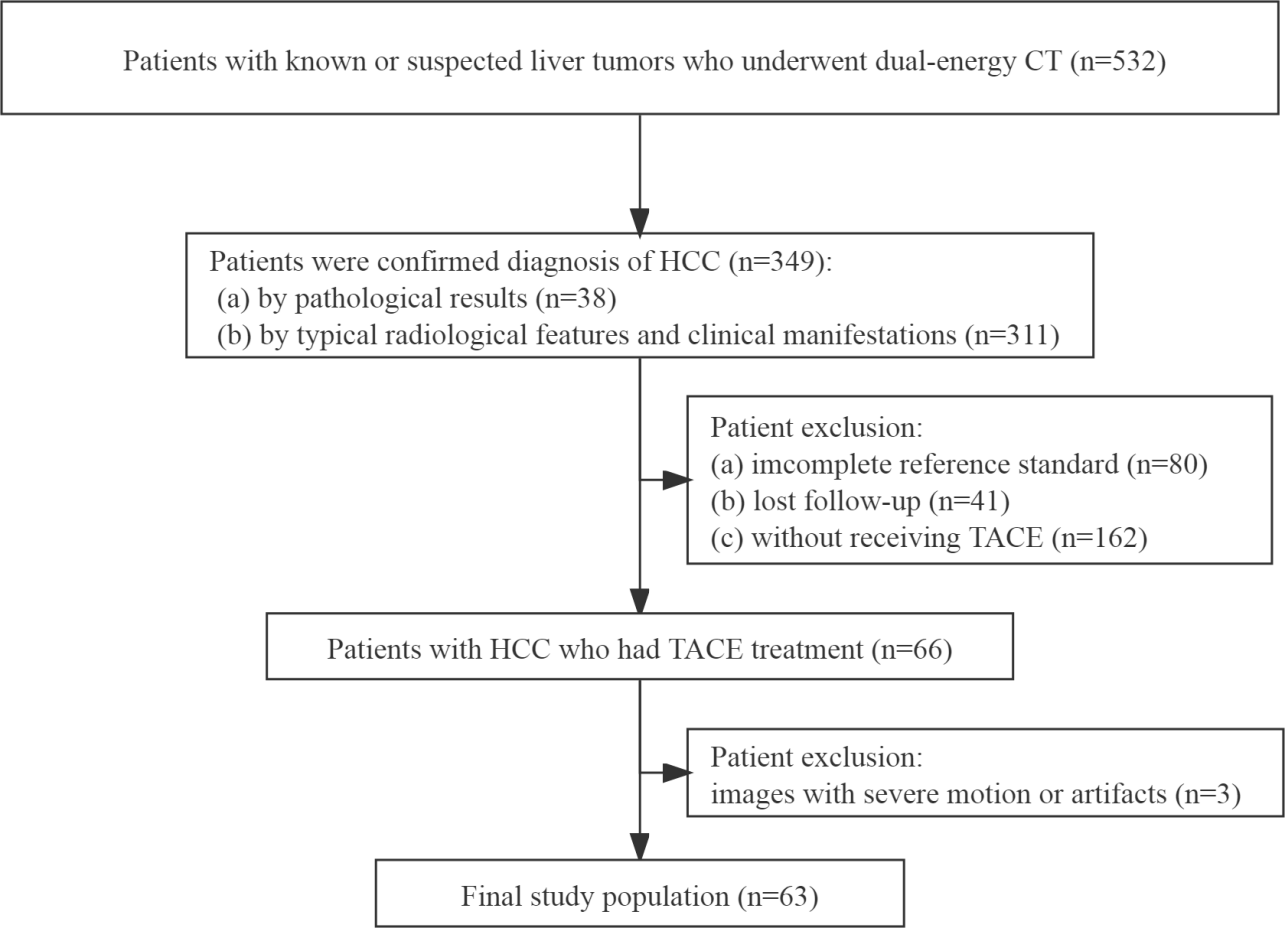
Flowchart of inclusion and exclusion criteria of our study population.

From May 2016 to December 2019, one radiologist with 5 years of experience in abdominal radiology reviewed our hospital’s radiology database and electronic medical records to identify patients who satisfied our eligibility criteria. The eligibility criteria were as follows: (a) patients were diagnosed with HCC, (b) patients with HCC underwent TACE treatment, and (c) patients underwent a baseline DECT scan before any treatment. Exclusion criteria were (a) an incomplete reference standard, (b) lost follow-up, and (c) CT images with severe motion or artifacts. The flowchart of the patient selection is displayed in **Figure 1**.

A study coordinator with three years of experience in abdominal radiology extracted data on the patient’s clinical and biochemical variables from electronic medical records, including age, gender, history of hepatitis, alcohol drinking history, diabetes mellitus, the level of alpha fetoprotein (AFP), and Child–Pugh classification.

### Reference standard

The tumors were diagnosed according to the pathological results, typical radiological features, and clinical manifestations.

Due to the atypical imaging characteristics, five individuals without cirrhosis were verified pathologically by CT or ultrasound-guided percutaneous core liver biopsy. For the other cases with classic characteristics for HCC, the diagnoses were established by consistent findings based on multiphasic contrast-enhanced CT images and high AFP levels (>11 ng/ml). The typical radiographic findings of HCC include non-rim arterial phase hyper-enhancement and non-peripheral washout appearance in the portal venous and/or equilibrium phases (EP).

The TACE in this article referred to conventional TACE, which involves mixing the chemotherapeutic drugs and embolic agents together and injecting through the feeding artery branch of the tumor (16). The detailed information was based on the patients’ comprehensive states.

Based on CT, MRI, and 18F-fluorodeoxyglucose-positron emission tomography, two other radiologists (with 10 and 7 years of experience in abdominal radiology, respectively) made a consensus statement in determining the number of tumors, the maximum diameter of the primary lesion, and the presence of vascular invasion and metastasis. In cases with multiple lesions, the largest one was considered to be the target lesion, and if at least one of them had an unclear boundary, the largest mensurable lesion was considered to be the target lesion. The presence of vascular invasion indicated that the vessel had an unclear boundary in imaging analysis. If the results of two observers were inconsistent, the decision would have been made by one other radiologist (with 20 years of experience in abdominal radiology). All observers were blinded to the treatment, tumor markers, and patient outcomes.

The modified Response Evaluation Criteria in Solid Tumors (mRECIST) was used to evaluate the treatment effect of HCC patients by using CT every 3 months. Overall survival (OS) was defined as the time from the baseline CT examination to death or the last follow-up, and the last follow-up was in March 2021. Progression-free survival (PFS) was defined as the time from the baseline CT examination to disease progression, death, or the latest follow-up examination, whichever occurred first. Although the survival outcomes of all patients were obtained, the PFS of six patients could not be evaluated due to incomplete follow-up CT examinations.

### CT imaging technique

Multiphasic enhanced CT examinations were performed on the Discovery CT scanner (GE Healthcare, Wisconsin, USA) in a cranio-caudal direction, with the scan range from the top of the diaphragm to the lower edge of the liver. Following the scout CT, all patients underwent unenhanced CT imaging in the conventional helical mode. After an intravenous injection of 1.5 ml/kg body weight of non-ionic contrast medium (Ioversol Injection, Optiray 320; Tyco Healthcare, Canada) at a fixed flowrate of 3.5 ml/s, the arterial phase scan began automatically 7 s after aortic enhancement exceeded 150 Hounsfield units (HU). The portal venous phase scan began 30 s after the arterial phase, and the scan delay for EP was set at 120 s after the portal venous phase.

Unenhanced, arterial phase and portal venous phase images were acquired using single-energy CT (SECT) with the following imaging parameters: tube voltage of 120 kVp, automatic tube current of 300–600 mA, collimation thickness of 0.625 mm, a standard soft-tissue kernel, and 5-mm-thick axial images reconstructed. The EP scan was obtained in dual-energy mode with a single tube and a fast-switching system between two peak voltage settings (80 and 140 kVp) and a tube current of 365 mA. The other scanning parameters were gantry rotation time of 0.6 s, helical pitch of 0.984:1, collimation thickness of 0.625 mm, acquisition slice thickness of 1.25 mm, and a matrix of 512 × 512. In order to balance image noise and spatial resolution, the reconstruction thickness and interval were both 5 mm. Decomposition images were suppressed by applying the adaptive statistical iterative reconstruction (ASIR) algorithm with standard 40% percentage.

### Image analysis

All images were transferred to a commercially available Advantage Workstation 4.6 (GE Healthcare) for quantitative analysis and were viewed using the Gemstone Spectral Imaging (GSI) volume viewer. Virtual monochromatic images (VMI) at 70 keV and iodine (water-paired) density images (IDI) were reconstructed from DECT for analyses. Two radiologists with more than 7 years of abdominal imaging experience, who were blinded to the clinical information, measured the CT value (HU) of the tumor and abdominal aorta in unenhanced phase and EP, and the iodine density (100 μg/ml) in EP. The value and iodine density measured by two radiologists were averaged to represent each region of interest (ROI).

**Figure 2:**
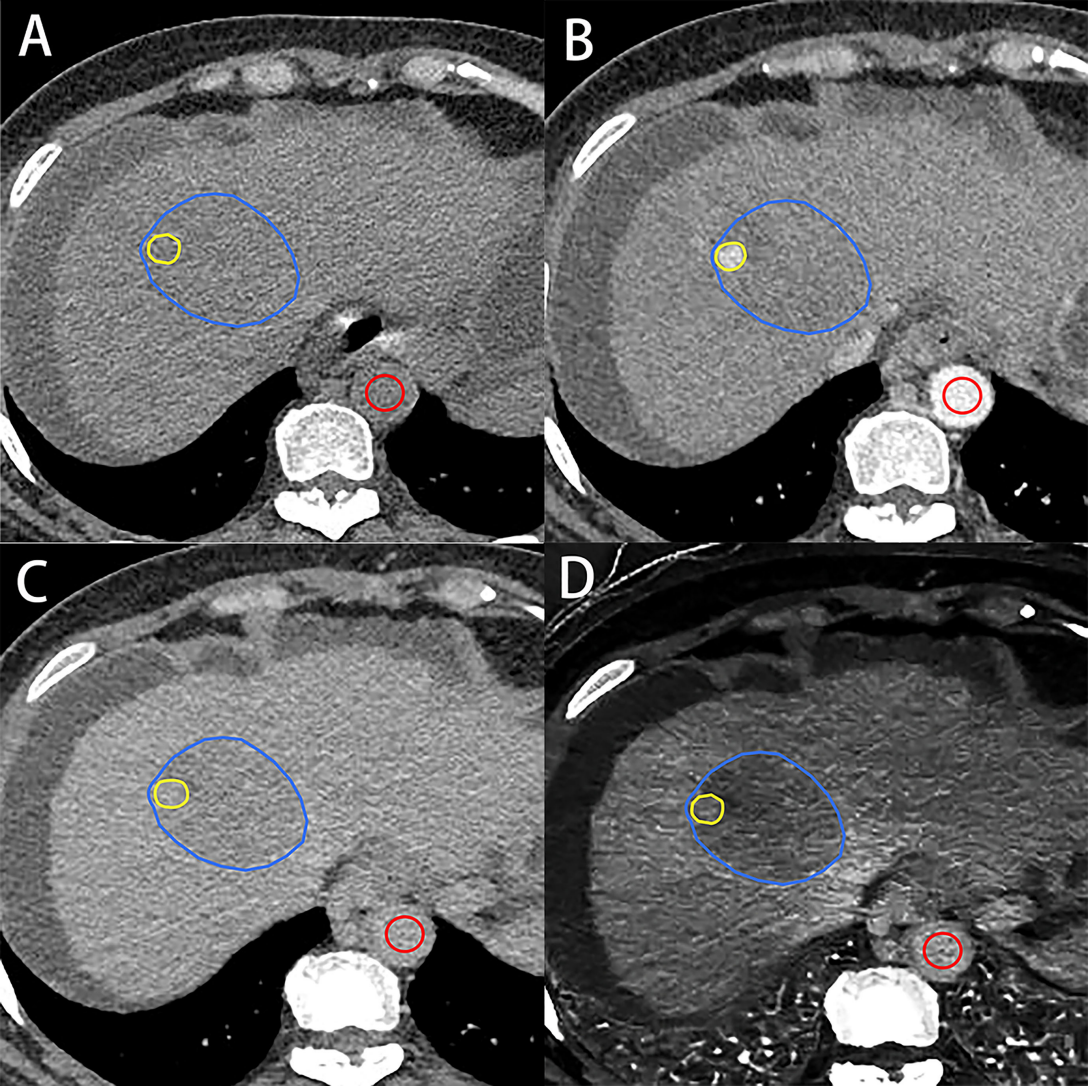
A 56-year-old woman with hepatocellular carcinoma. Axial unenhanced **(A)**, arterial **(B)** and equilibrium-phase contrast-enhanced **(C)**, and iodine density **(D)** images show the regions of interest on the tumor. Regions of interest on the tumor were manually outlined along the boundaries of target lesion (blue irregular shape) and the hyperenhancing portion of the lesion (yellow irregular shape) and aorta (red circle shape). VMI- and IDI-fECV based on the whole tumor and the maximum enhancement of the tumor were 34.87% and 44.13% (Whole) and 36.16% and 46.51% (Maximum), respectively.

Using an electronic cursor and mouse, the ROIs of the whole tumor (Whole) were manually outlined layer by layer along the boundaries of the target lesion, including necrotic areas, and then averaged. Window width and window level could be adjusted and/or combined enhanced images if the tumor boundaries were obscure. According to the maximum enhancement part of the tumor in the arterial phase, the ROI of the maximum enhancement (Maximum) part of the tumor in EP was drawn by using the copy–paste tool with minor adjustments (**Figure 2**). When multiple similar enhancements were shown in the arterial phase, the largest hyperenhancing portion of the target lesion was selected.

The formulas for calculating fECV based on the whole tumor and the maximum enhancement part of the tumor were as follows:


$$\text{VMI}‐\text{fECV}\left(\%\right)\;=\;\left(1‐\text{hematocrit}\right)\times (\Delta {\text{HU}}_{\text{tumor}}/\Delta {\text{HU}}_{\text{aorta}})\;\times \;100$$

$$\text{IDI}‐\text{fECV}\left(\%\right)\;=\;\left(1‐\text{hematocrit}\right)\times {\text{(ID}}_{\text{tumor}}/{\text{ID}}_{\text{aorta}}\text{) }\times \;100$$

where ΔHU are the differences in Hounsfield units between the EP and unenhanced phases. ID means iodine density in EP.

### Statistical analysis

All statistical analyses were performed using SPSS (version 21; Chicago, IL, USA). The intraclass correlation coefficient (ICC) was assessed to evaluate reproducibility among the measurements of VMI- and IDI-fECV. The Pearson correlation coefficient and Bland–Altman analysis were used to determine the correlation between VMI- and IDI-fECV. All the clinical, biochemical, and imaging variables on PFS and OS were analyzed with univariate and multivariate analyses using Cox proportional hazards models. Variables with *p*-values< 0.10 after univariate analysis were analyzed in multivariate analysis. A *p-*value< 0.05 was considered statistically significant. To determine optimal cutoff values of tumor fECV for predicting OS ≥ 1 year and PFS ≥ 6 months in HCC patients, receiver operating characteristic curve (ROC) analyses were performed. The areas under the curve (AUC) of 0.5–0.7, 0.7–0.9, and above 0.9 indicate low diagnostic value, medium diagnostic value, and high diagnostic value, respectively.

To assess OS and PFS in subjects with tumor fECV above and below the optimal ROC-derived cutoff values, the Kaplan–Meier method with the log-rank test was applied to the survival curve.

## Results

### Patient characteristics and radiation exposure

A total of 63 patients were included in our study, with 46 men (age range, 30–68 years old; mean age of 54 years old) and 17 women (age range, 48–79 years old; mean age of 49 years old). The maximum diameter of tumor ranges from 35 to 156 mm. A total of 11 patients with multiple lesions and eight patients with Child–Pugh grade C had tumor stroke. Metastasis occurred in approximately 19% patients including intrahepatic and extrahepatic metastasis according to the auxiliary image examination.

The median OS and PFS durations were 15.1 months (95% confidence intervals (CI) = 12.8–17.4) and 11.5 months (95% CI = 9.4–13.8), respectively.

The mean CT dose index volumes for unenhanced phase and EP were 16.2 mGy (range, 13.3–17.1 mGy) and 11.3 mGy, respectively, and that of dual-phase enhanced CT was 28.1 mGy (range, 26.7–34.1 mGy).

### Tumor fECV

The average CT-Whole and CT-Maximum in EP were 73.66 ± 12.19 HU (range, 32.62–86.14 HU) and 80.96 ± 13.36 HU (range, 53.13–97.42 HU), while the average VMI-fECV-Whole and the VMI-fECV-Maximum of the HCC were 22.48% ± 10.17% (range, 0.65%–43.26%) and 27.05% ± 9.67% (range, 10.41%–48.23%), respectively. The average I-Whole and I-Maximum in EP were 13.75 ± 9.68 100 μg/ml (range, 3.65–31.80 100 μg/ml) and 19.42 ± 9.83 100 μg/ml (range, 9.35–36.60 100 μg/ml), while the average IDI-fECV-Whole and the IDI-fECV-Maximum of the HCC were 27.64% ± 10.32% (range, 5.72%–49.14%) and 33.94% ± 9.82% (range, 14.64%–55.85%), respectively. The fECV-Whole was significantly lower than the fECV-Maximum determined by virtual SECT (*p* = 0.000) and DECT (*p* = 0.000) respectively.

**Figure 3:**
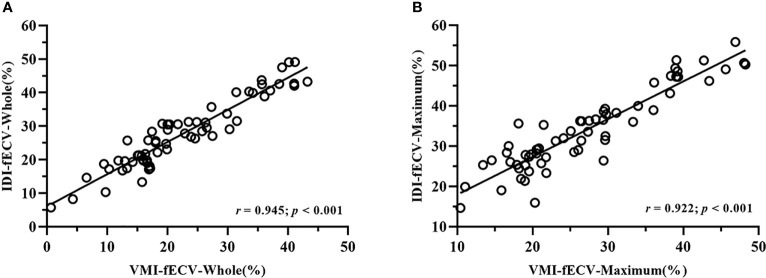
Positive linear correlation between VMI-fECV-Whole and IDI-fECV-Whole **(A)** and VMI-fECV-Maximum and IDI-fECV-Maximum **(B)**.

**Figure 4:**
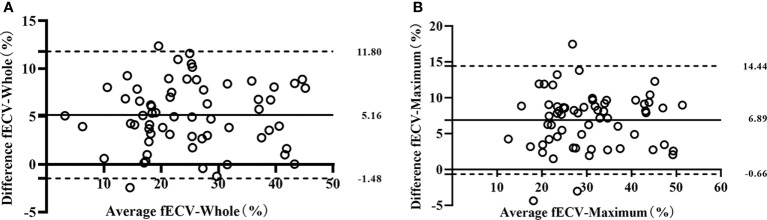
Bland–Altman plots for VMI-fECV-Whole and IDI-fECV-Whole **(A)** and VMI-fECV-Maximum and IDI-fECV-Maximum **(B)**. The solid line stands for mean bias, and the lower and upper dotted lines stand for 95% limits of agreement.

Interobserver agreement was good; ICCs for the VMI-fECV-Whole and VMI-fECV-Maximum were 0.83 (95% CI = 0.79–0.85) and 0.89 (95% CI = 0.84–0.92), respectively, and that for IDI-fECV-Whole and IDI-fECV-Maximum were 0.90 (95% CI = 0.83–0.93) and 0.94 (95% CI = 0.91–0.96), respectively. The correlations between the VMI- and IDI-fECV were strong (*p*< 0.001; **Figure 3**). Bland–Altman plots of the VMI- and IDI-fECV are presented in **Figure 4**. A bias was obtained (5.16%) toward higher fECV as determined by IDI-fECV-Whole with 95% CI of −1.48% and 11.80% (**Figure 4A**), while the bias was 6.89% as determined by IDI-fECV-Maximum with 95% CI of −0.66% and 14.44% (**Figure 4B**). The bias between VMI-fECV-Whole and IDI-fECV-Whole methods was 5.16% (95% CI = −1.48%–11.80%), which was slightly lower than that of VMI-fECV-Maximum and IDI-fECV-Maximum (6.89%; 95% CI = −0.66%–14.44%).

### Correlation of parameters with OS and PFS

The results of univariate and multivariate analyses on OS and PFS are presented in **Tables 1** and **2**. Gender (OS, *p* = 0.023; PFS, *p* = 0.039), tumor number (OS, *p* = 0.014; PFS, *p* = 0.007), maximum diameter of the primary lesion (OS, *p* = 0.000; PFS, *p* = 0.000), the level of AFP (OS, *p* = 0.002; PFS, *p* = 0.011), Child–Pugh classification (OS, *p* = 0.008; PFS, *p* = 0.039), the presence of metastasis (OS, *p* = 0.000; PFS, *p* = 0.000), and vascular invasion (OS, *p* = 0.000; PFS, *p* = 0.006) showed significant influence on OS and PFS in the univariate analysis. Lower tumor CT and iodine density and VMI- and IDI-fECV based on the Whole tumor and the Maximum enhancement part of the tumor determined by virtual SECT and DECT in EP were associated with poor OS and PFS (all *p*< 0.05).

**Table 1:** Univariate and multivariate Cox regression analyses on overall survival in patients with HCC.

Variable	Patients	Univariate analysis		Multivariate analysis	
		Hazard ratio	*p*	Hazard ratio	*p*
Age (years)		0.997 (0.963–1.032)	0.862	NA	
Gender: male vs female	46 vs 17	2.189 (1.114–4.302)	0.023	1.468 (0.330–6.529)	0.614
Tumor number Multiple vs single	11 vs 52	2.350 (1.189–4.647)	0.014	2.205 (0.835–5.821)	0.110
Maximum diameter (cm)		1.020 (1.013–1.028)*	0.000	1.017 (1.005–1.030)*	0.008
Hepatitis Present vs absent	47 vs 16	1.475 (0.756–2.879)	0.255	NA	
Alcohol drinking Present vs absent	49 vs 14	1.968 (0.955–4.056)	0.066	1.077 (0.263–4.410)	0.918
Diabetes mellitus Present vs absent	31 vs 32	1.091 (0.629–1.893)	0.757	NA	
AFP (ng/ml)		1.000 (1.000–1.000)*	0.002	1.000 (1.000–1.000)	0.097
Child–Pugh grade C *vs*. A	8 vs 28	3.860 (1.621–9.190)	0.002	4.506 (1.450–12.002)	0.009
Metastasis: Present vs absent	12 vs 51	6.033 (4.089–11.953)	0.000	3.844 (1.364–10.834)	0.011
Vascular invasion Present vs absent	17 vs 46	3.776 (2.012–7.084)	0.000	1.601 (0.686–3.737)	0.276
CT-Whole-EP (HU)		0.972 (0.958–0.986)*	0.000	NA	
CT-Maximum-EP (HU)		0.972 (0.957–0.988)*	0.000	NA	
I-Whole-EP (100 μg/ml)		0.948 (0.919–0.978)*	0.001	0.961 (0.929–1.213)	0.380
I-Maximum-EP (100 μg/ml)		0.945 (0.917–0.974)*	0.000	0.975 (0.940–1.190)	0.166
VMI-fECV-Whole (%)		0.932 (0.905–0.960)*	0.000	NA	
VMI-fECV-Maximum (%)		0.914 (0.883–0.947)*	0.000	NA	
IDI-fECV-Whole (%)		0.941 (0.916–0.968)*	0.000	0.947 (0.914–1.324)	0.061
IDI-fECV-Maximum (%)		0.904 (0.871–0.938)*	0.000	0.910 (0.844–0.962)*	0.000

Data in parentheses denote the 95% confidence intervals.

*Hazard ratio per unit increase.

AFP, alpha fetoprotein; EP, equilibrium phase; fECV, extracellular volume fraction; VMI, virtual monochromatic images; IDI, iodine density images; NA, not applicable due to the variables with p-values > 0.10 in univariate analysis or in order to reduce the multicollinearity between the virtual SECT and DECT.

**Table 2:** Univariate and multivariate Cox regression analyses on progression-free survival in patients with HCC.

Variable	Patients	Univariate analysis		Multivariate analysis	
		Hazard ratio	*p*	Hazard ratio	*p*
Age (years)		0.993 (0.957–1.031)	0.719	NA	
Gender: male vs female	42 vs 15	2.174 (1.040–4.545)	0.039	1.252 (0.247–6.345)	0.786
Tumor number Multiple vs single	11 vs 46	2.746 (1.317–5.722)	0.007	2.284 (1.028-5.491)	0.045
Maximum diameter (cm)		1.017 (1.010–1.025)*	0.000	1.008 (0.994–1.023)	0.255
Hepatitis Present vs absent	41 vs 16	1.372 (0.693–2.717)	0.364	NA	
Alcohol drinking Present vs absent	45 vs 12	2.007 (0.894–4.509)	0.092	1.202 (0.287–5.033)	0.801
Diabetes mellitus Present vs absent	29 vs 28	1.072 (0.596–1.929)	0.816	NA	
AFP (ng/ml)		1.000 (1.000–1.000)*	0.011	1.000 (1.000–1.000)	0.527
Child–Pugh grade C vs A	8 vs 27	2.794 (1.179–6.618)	0.020	2.823 (0.865–9.218)	0.086
Metastasis Present vs absent	12 vs 45	4.889 (2.256–10.592)	0.000	2.442 (0.928–6.427)	0.071
Vascular invasion Present vs absent	17 vs 40	2.431 (1.285–4.599)	0.006	1.145 (0.312–1.776)	0.506
CT-Whole-EP (HU)		0.976 (0.961–0.991)*	0.002	NA	
CT-Maximum-EP (HU)		0.977 (0.961–0.993)*	0.004	NA	
I-Whole-EP (100 μg/ml)		0.954 (0.922–0.987)*	0.007	0.981 (0.970–1.344)	0.112
I-Maximum-EP (100 μg/ml)		0.949 (0.918–0.982)*	0.002	0.987 (0.866–1.125)	0.846
VMI-fECV-Whole (%)		0.940 (0.912–0.970)*	0.000	NA	
VMI-fECV-Maximum (%)		0.923 (0.889–0.959)*	0.000	NA	
IDI-fECV-Whole (%)		0.949 (0.921–0.977)*	0.000	0.968 (0.913–1.250)	0.411
IDI-fECV-Maximum (%)		0.917 (0.883–0.953)*	0.000	0.935 (0.911-0.980)*	0.028

Data in parentheses denote the 95% confidence intervals.

*Hazard ratio per unit increase.

AFP, alpha fetoprotein; EP, equilibrium phase; fECV, extracellular volume fraction; VMI, virtual monochromatic images; IDI, iodine density images; NA, not applicable due to the variables with p values > 0.10 in univariate analysis or in order to reduce the multicollinearity between the virtual SECT and DECT.

**Figure 5:**
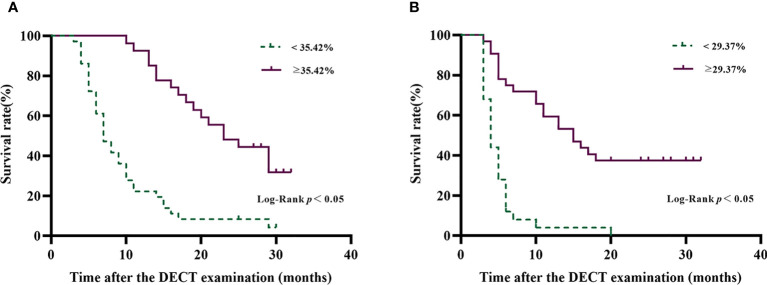
Overall survival curves **(A)** and progression-free survival curves **(B)** of patients with HCC after TACE disaggregated by tumor IDI-fECV-Maximum. The optimal ROC-derived cutoff values were 35.42% and 29.37% on OS and PFS, respectively. Patients with above the tumor IDI-fECV-Maximum have a relatively longer survival time than those with below tumor IDI-fECV-Maximum.

In order to reduce the multicollinearity between the virtual SECT and DECT, the CT value and VMI-fECV derived from the virtual SECT were excluded from multivariate analysis. The tumor IDI-fECV-Maximum was the only congruent independent predictor for HCC patients after TACE in the multivariate analysis on OS (*p* = 0.000) and PFS (*p* = 0.028). In addition, multivariate analysis also showed that tumor number was a significant predictor of PFS (*p* = 0.045), but not OS (*p* = 0.110). On the contrary, the maximum diameter, the presence of metastasis, and Child–Pugh C were significant predictors of OS (*p* = 0.008, 0.011, and 0.009, respectively) but not PFS (*p* = 0.255, 0.071, and 0.086, respectively). The AUCs of the IDI-fECV-Maximum for predicting OS ≥ 1 year and PFS ≥ 6 months were 0.881 (95% CI = 0.793–0.969) and 0.800 (95% CI = 0.683–0.918), respectively (**Supplementary Figure S1**). The optimal cutoff values of tumor fECV were 35.42% and 29.37% on OS and PFS, respectively; the sensitivity was 76% and 86%, respectively; and the specificity was 70% and 60%, respectively. Patients with higher tumor fECV showed better OS and PFS than those with lower tumor fECV (*p*< 0.05) when stratified by the cutoff values (**Figure 5**).

## Discussion

Our study demonstrated that fECV, determined by IDI generated from equilibrium contrast-enhanced DECT, is a non-invasive evaluation method for predicting the survival outcomes of HCC patients treated with TACE. Compared with fECV determined by VMI, fECV determined by IDI undoubtedly decreases the radiation dose by 30% with EP alone and improves work efficiency.

The IDI-fECV correlated well with the VMI-fECV in our study, which is consistent with previous research reports (17–19), suggesting that the IDI-fECV may be similar to the VMI-fECV used for tumor prognostic analysis. Previous studies have reported that the fECV based on unenhanced and equilibrium phases has been widely used for the assessment of liver fibrosis (13, 20–22). An additional study found that iodine uptake of the fECV had a strong positive correlation with liver fibrosis by using an iodine (water-paired) density image without the need for a subtraction process (23). In addition, another key aspect of this study was that the IDI-fECV was higher than the VMI-fECV, which was in accordance with the previous pancreatic ductal adenocarcinoma study (24). The reason may be that the IDI generated from DECT can quantitatively and intuitively evaluate the distribution of iodine in tissues, and the iodine density estimates will be more precise, which represents the true proportion or relative quantity of iodine and has been widely used in tumor diagnosis and curative effect evaluation (25–28).

Notably, the fECV-Whole was lower than the fECV-Maximum no matter whether determined by virtual SECT or DECT. This observation is in agreement with the reported heterogeneity of tumors within cancers, which can be interpreted as the malignant tumor growing rapidly beyond tumor vascular nutrition, resulting in poorly organized cell structure (28). It indirectly reflects the blood supply of the tumor, which is the key determinant of tumor biological aggressiveness, tumor interventional therapy, and prognosis. We adopted the tumor fECV-Maximum mainly on the basis of the mRECIST standard, in which the survival tumor is considered to be the evaluated subject and can better reflect the survival tumor burden in HCC patients after intervention and targeted drug treatment. While the tumor fECV-Whole can better reflect the overall biological behavior because of the tumor heterogeneity.

Based on tumor IDI-fECV-Maximum, patients with higher fECV have a relatively longer survival time than those with lower fECV, which is consistent with the results of Fukukura et al. (24). Moreover, the tumor IDI-fECV-Maximum demonstrated a relatively higher diagnostic value than other variables from multivariate analysis for predicting OS ≥ 1 year and PFS ≥ 6 months. The cutoff value calculated by the ROC curve was used as a reference, and the OS and PFS survival curves were drawn with obvious differences. We speculate that tumors with higher extravascular volume fractions have higher sensitivity and an enormous demand for blood supply. Once the blood supply is blocked by TACE, the effect will be more pronounced, leading to a better treatment outcome.

The DECT system in the present study with a single detector and single source has distinct advantages for the measurement of iodine retention and other forms of dual energy. The 70 keV VMI in the present study were chosen, since prior studies (29, 30) have shown that images at a 70 keV energy level are equivalent to 120 kVp images. In the measurement of iodine density, iodine can be more freely distributed to the plasma and extracellular space than a macromolecular contrast agent. Hepatocytes are capable of neglecting intracellular uptake, and the ratio of the difference between the tumor and the aorta in EP represents the proportion of mesenchyme, which can offset the microvascular part to a certain extent. Although the most accurate EP is 30 min, the 180-s EP can be easily integrated into routine clinical examinations to remove biliary interference (31). Whether different equilibration scanning times can lead to different results or not, it will require a substantial amount of time to verify the influence on the fECV.

Our findings also showed that male sex, multiple lesions, a longer diameter, a higher AFP level, the presence of metastasis and vascular invasion, and Child–Pugh C were risk factors for OS and PFS in univariate analysis, which was consistent with previous findings (32, 33). Furthermore, the importance of CT value based on the CT images and the iodine density based on the IDI to evaluate the survival outcomes of HCC patients in univariate analysis was also identified, even though they had no statistical significance in multivariate analysis. Nonetheless, whether standardized CT value and iodine density have statistical significance or not for the prognosis evaluation of HCC patients still needs to be validated.

There were several limitations to our study. First, the number of patients who met the screening criteria was relatively small, many patients did not have pathological diagnoses, and the TACE procedures were not completely consistent because of the tumor characteristics and patients’ general condition. Independent large-sample trials with pathological diagnoses and consistent TACE procedures in the future are needed to validate these results. Second, various factors affect the iodine density of tumors, including the tumor’s microvascular environment, scanning technology, and physiological changes caused by other concurrent diseases. However, we extended to the maximum enhancement part of the tumor and tried to refine the composition ratio to a certain extent to reduce the result bias caused by the tumor characteristics. Third, our results were gained from a single-source DECT using a rapid-kVp switching system, and 70 keV VMI in EP was used instead of conventional 120 kVp images. Of note, whether these equivalent images can completely replace the traditional images still needs further study.

## Conclusion

Our study retrospectively confirmed that the fECV derived from IDI showed a good correlation with the fECV derived from 70 keV VMI. The IDI-fECV-Maximum demonstrated the potential possibility of predicting the survival outcomes of HCC patients after TACE, representing a new potential approach toward the clinical evaluation of HCC.

## Data availability statement

The original contributions presented in the study are included in the article/**Supplementary Material**. Further inquiries can be directed to the corresponding authors.

## Ethics statement

Ethical review and approval was not required for the study on human participants in accordance with the local legislation and institutional requirements. Written informed consent for participation was not required for this study in accordance with the national legislation and the institutional requirements.

## Author contributions

YC and PL designed this work. YC, KS, ZL, HW, NL, PZ, XL, BS, and PH integrated and analyzed the data. YC, KS, and PL wrote this manuscript. YC, KS, PL, and JG edited and revised the manuscript. All authors contributed to the article and approved the submitted version.

## References

[1] Valery PC Laversanne M Clark PJ Petrick JL McGlynn KA Bray F. Projections of primary liver cancer to 2030 in 30 countries worldwide. Hepatology (2018) 67:600–11. doi: 10.1002/hep.29498 PMC5832532 28859220

[2] McGlynn KA London WT. Epidemiology and natural history of hepatocellular carcinoma. Best Pract Res Clin Gastroenterol (2005) 19:3–23. doi: 10.1016/j.bpg.2004.10.004 15757802

[3] Shen Y Guo H Wu T Lu Q Nan KJ Lv Y. Lower education and household income contribute to advanced disease, less treatment received and poorer prognosis in patients with hepatocellular carcinoma. J Cancer (2017) 8:3070–7. doi: 10.7150/jca.19922 PMC5604458 28928898

[4] Raoul JL Forner A Bolondi L Cheung TT Kloeckner R de Baere T. Updated use of TACE for hepatocellular carcinoma treatment: how and when to use it based on clinical evidence. Cancer Treat Rev (2019) 72:28–36. doi: 10.1016/j.ctrv.2018.11.002 30447470

[5] Shim JH Lee HC Kim SO Shin YM Kim KM Lim YS. Which response criteria best help predict survival of patients with hepatocellular carcinoma following chemoembolization? a validation study of old and new models. Radiology (2012) 262:708–18. doi: 10.1148/radiol.11110282 22187634

[6] Llovet JM Real MI Montaña X Planas R Coll S Aponte J. Arterial embolisation or chemoembolisation versus symptomatic treatment in patients with unresectable hepatocellular carcinoma: a randomised controlled trial. Lancet (2002) 359:1734–9. doi: 10.1016/S0140-6736(02)08649-X 12049862

[7] Chen M Cao J Hu J Topatana W Li S Juengpanich S. Clinical-radiomic analysis for pretreatment prediction of objective response to first transarterial chemoembolization in hepatocellular carcinoma. Liver Cancer (2021) 10:38–51. doi: 10.1159/000512028 33708638 PMC7923935

[8] Yue X Jiang Q Hu X Cen C Song S Qian K. Quantitative dual-energy CT for evaluating hepatocellular carcinoma after transarterial chemoembolization. Sci Rep (2021) 11:11127. doi: 10.1038/s41598-021-90508-9 34045528 PMC8160271

[9] Lin YM Chiou YY Wu MH Huang SS Shen SH. Postablation assessment of hepatocellular carcinoma using dual-energy CT: comparison of half versus standard iodine contrast medium. PloS One (2019) 14:e0219577. doi: 10.1371/journal.pone.0219577 31287838 PMC6615706

[10] Xing G Wang S Li C Zhao X Zhou C. [Value of quantitative iodine-based material decomposition images with gemstone spectral CT imaging in the follow-up of patients with hepatocellular carcinoma after TACE treatment]. Zhonghua Zhong Liu Za Zhi (2015) 37:208–12. 25975791

[11] Thaiss WM Haberland U Kaufmann S Hepp T Schulze M Blum AC. Dose optimization of perfusion-derived response assessment in hepatocellular carcinoma treated with transarterial chemoembolization: comparison of volume perfusion CT and iodine concentration. Acad Radiol (2019) 26:1154–63. doi: 10.1016/j.acra.2018.09.026 30482626

[12] Bak S Kim JE Bae K Cho JM Choi HC Park MJ. Quantification of liver extracellular volume using dual-energy CT: utility for prediction of liver-related events in cirrhosis. Eur Radiol (2020) 30:5317–26. doi: 10.1007/s00330-020-06876-9 32335746

[13] Ito E Sato K Yamamoto R Sakamoto K Urakawa H Yoshimitsu K. Usefulness of iodine-blood material density images in estimating degree of liver fibrosis by calculating extracellular volume fraction obtained from routine dual-energy liver CT protocol equilibrium phase data: preliminary experience. Jpn J Radiol (2020) 38:365–73. doi: 10.1007/s11604-019-00918-z 31907717

[14] Sofue K Tsurusaki M Mileto A Hyodo T Sasaki K Nishii T. Dual-energy computed tomography for non-invasive staging of liver fibrosis: accuracy of iodine density measurements from contrast-enhanced data. Hepatol Res (2018) 48:1008–19. doi: 10.1111/hepr.13205 29908040

[15] Hong S Kim JE Cho JM Choi HC Won JH Na JB. Quantification of liver extracellular volume using dual-energy CT for ruling out high-risk varices in cirrhosis. Eur J Radiol (2022) 148:110151. doi: 10.1016/j.ejrad.2022.110151 35032849

[16] Department of Medical Administration, National Health and Health Commission of the People's Republic of China. Guidelines for diagnosis and treatment of primary liver cancer in China (2019 edition). Zhonghua Gan Zang Bing Za Zhi (2020) 28:112–28. doi: 10.3760/cma.j.issn.1007-3418.2020.02.004 PMC12769107 32164061

[17] Fukukura Y Kumagae Y Higashi R Hakamada H Nakajo M Maemura K. Extracellular volume fraction determined by equilibrium contrast-enhanced dual-energy CT as a prognostic factor in patients with stage IV pancreatic ductal adenocarcinoma. Eur Radiol (2020) 30:1679–89. doi: 10.1007/s00330-019-06517-w 31728691

[18] Ohta Y Kitao S Watanabe T Mukai N Kishimoto J Yamamoto K. Measurement of myocardial extracellular volume fraction from iodine density images using single-source, dual-energy computed tomography: a feasibility study. J Comput Assist Tomogr (2017) 41:750–6. doi: 10.1097/RCT.0000000000000587 28240638

[19] Zhou Y Geng D Su GY Chen XB Si Y Shen MP. Extracellular volume fraction derived from dual-layer spectral detector computed tomography for diagnosing cervical lymph nodes metastasis in patients with papillary thyroid cancer: a preliminary study. Front Oncol (2022) 12:851244. doi: 10.3389/fonc.2022.851244 35756662 PMC9213667

[20] Ozaki K Ishida T Ohtani T Shimada M Kimura H Gabata T. Assessing the progression of segmental fibrosis in chronic liver disease using extracellular volume fractions. Eur J Radiol (2021) 145:110033. doi: 10.1016/j.ejrad.2021.110033 34808581

[21] Marri UK Das P Shalimar Kalaivani M Srivastava DN Madhusudhan KS. Noninvasive staging of liver fibrosis using 5-minute delayed dual-energy CT: comparison with US elastography and correlation with histologic findings. Radiology (2021) 298:600–8. doi: 10.1148/radiol.2021202232 33399510

[22] Guo SL Su LN Zhai YN Chirume WM Lei JQ Zhang H. The clinical value of hepatic extracellular volume fraction using routine multiphasic contrast-enhanced liver CT for staging liver fibrosis. Clin Radiol (2017) 72:242–6. doi: 10.1016/j.crad.2016.10.003 28341030

[23] Wada N Fujita N Ishimatsu K Takao S Yoshizumi T Miyazaki Y. A novel fast kilovoltage switching dual-energy computed tomography technique with deep learning: utility for non-invasive assessments of liver fibrosis. Eur J Radiol (2022) 155:110461. doi: 10.1016/j.ejrad.2022.110461 35970119

[24] Fukukura Y Kumagae Y Higashi R Hakamada H Takumi K Maemura K. Extracellular volume fraction determined by equilibrium contrast-enhanced multidetector computed tomography as a prognostic factor in unresectable pancreatic adenocarcinoma treated with chemotherapy. Eur Radiol (2019) 29:353–61. doi: 10.1007/s00330-018-5570-4 29922930

[25] Lv P Lin XZ Chen K Gao J. Spectral CT in patients with small HCC: investigation of image quality and diagnostic accuracy. Eur Radiol (2012) 22:2117–24. doi: 10.1007/s00330-012-2485-3 22618521

[26] Lin XZ Wu ZY Tao R Guo Y Li JY Zhang J. Dual energy spectral CT imaging of insulinoma-value in preoperative diagnosis compared with conventional multi-detector CT. Eur J Radiol (2012) 81:2487–94. doi: 10.1016/j.ejrad.2011.10.028 22153746

[27] Noda Y Goshima S Miyoshi T Kawada H Kawai N Tanahashi Y. Assessing chemotherapeutic response in pancreatic ductal adenocarcinoma: histogram analysis of iodine concentration and CT number in single-source dual-energy CT. AJR Am J Roentgenol (2018) 211:1221–6. doi: 10.2214/AJR.18.19791 30332288

[28] Noid G Godfrey G Hall W Shah J Paulson E Knechtges P. Predicting treatment response from extracellular volume fraction for chemoradiation therapy of pancreatic cancer. Int J Radiat Oncol Biol Phys (2023) 115:803–8. doi: 10.1016/j.ijrobp.2022.09.084 36210026

[29] Rodriguez-Granillo GA Carrascosa P Cipriano S de Zan M Deviggiano A Capunay C. Myocardial signal density levels and beam-hardening artifact attenuation using dual-energy computed tomography. Clin Imaging (2015) 39:809–14. doi: 10.1016/j.clinimag.2015.04.007 25935519

[30] So A Lee TY Imai Y Narayanan S Hsieh J Kramer J. Quantitative myocardial perfusion imaging using rapid kVp switch dual-energy CT: preliminary experience. J Cardiovasc Comput Tomogr (2011) 5:430–42. doi: 10.1016/j.jcct.2011.10.008 22146502

[31] Van Beers BE Leconte I Materne R Smith AM Jamart J Horsmans Y. Hepatic perfusion parameters in chronic liver disease: dynamic CT measurements correlated with disease severity. AJR Am J Roentgenol (2001) 176:667–73. doi: 10.2214/ajr.176.3.1760667 11222202

[32] Lee YH Hsu CY Huang YH Su CW Lin HC Hsia CY. α-fetoprotein-to-total tumor volume ratio predicts post-operative tumor recurrence in hepatocellular carcinoma. J Gastrointest Surg (2013) 17:730–8. doi: 10.1007/s11605-012-2081-5 23188220

[33] Nagaoki Y Hyogo H Aikata H Tanaka M Naeshiro N Nakahara T. Recent trend of clinical features in patients with hepatocellular carcinoma. Hepatol Res (2012) 42:368–75. doi: 10.1111/j.1872-034X.2011.00929.x 22151896

