# Supplementary material for: Survival prediction of hepatocellular carcinoma by measuring the extracellular volume fraction with single-phase contrast-enhanced dual-energy CT imaging
Source: Front Oncol. 2023 Jul 19;13:1199426. doi: 10.3389/fonc.2023.1199426 (PMC10394647; doi:10.3389/fonc.2023.1199426)
Supplement: Supplementary file 1 [file Image_1.pdf]

Supplementary Material

1 Supplementary Figures

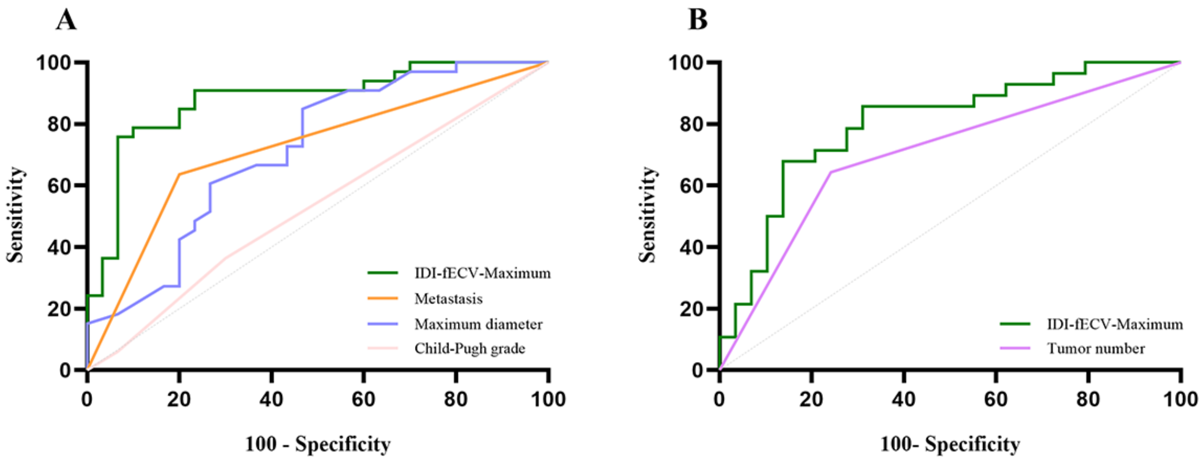

Supplementary Figure 1. ROC curves of multivariate Cox regression analyses on overall survival (A) and progression-free survival (B) in patients with HCC. The AUCs of the significant variables on OS were 0.881 for IDI-fECV-Maximum, 0.718 for Metastasis, 0.715 for Maximum diameter, and 0.529 for Child-Pugh grade, respectively. The AUCs of the significant variables on PFS were 0.800 for IDI-fECV-Maximum and 0.701 for Tumor number.
